# Nursing Home Social Services Directors' Roles and Self-Efficacy in Suicide Risk Management

**DOI:** 10.1093/geroni/igab046.447

**Published:** 2021-12-17

**Authors:** Xiaochuan Wang, Kelsey Simons, Denise Gammonley, Amy Restorick Roberts, Mercedes Bern-Klug

**Affiliations:** 1 University of Central Florida, Orlando, Florida, United States; 2 University of Rochester School of Medicine & Dentistry, Department of Psychiatry, Rochester, New York, United States; 3 Miami University, Oxford, Ohio, United States; 4 University of Iowa, Iowa City, Iowa, United States

## Abstract

Nursing home (NH) residents face many risk factors for late life suicide, and transitions into and out of NHs represent risk periods for suicide. Based on data from the 2019 National Nursing Home Social Services Directors survey (n = 924), this presentation describes NH social services directors (SSDs) roles in managing suicide risk and factors that influence self-efficacy in this area. Nearly one-fifth (19.7%) of SSDs lack of self-efficacy in suicide risk management, reporting needing significant preparation time or being not able to train others on this topic. Results of ordinal logistic regression indicate that SSDs who consider insufficient social services staffing as a minor barrier (comparing with a major barrier) to psychosocial care, those who report greater involvement in safety planning for suicide risk, and those with Master’s degree, are more likely to perceive greater self-efficacy in suicide risk management. Implications for training and staffing will be discussed.

